# Implication of Human Endogenous Retrovirus Envelope Proteins in Placental Functions

**DOI:** 10.3390/v6114609

**Published:** 2014-11-24

**Authors:** Adjimon Gatien Lokossou, Caroline Toudic, Benoit Barbeau

**Affiliations:** Département des Sciences Biologiques and Centre de recherche BioMed, Université du Québec à Montréal, 2080 Saint-Urbain, Montréal, PQ H2X 3X8, Canada; E-Mails: gatien.lokossou@gmail.com (A.G.L.); caroline.toudic22@gmail.com (C.T.)

**Keywords:** human endogenous retrovirus, Syncytin-1 and -2, placenta, syncytiotrophoblast, immunotolerance, exosomes, pre-eclampsia

## Abstract

Human endogenous retroviruses (ERVs) represent 8% of the total human genome. Although the majority of these ancient proviral sequences have only retained non-coding long terminal repeats (LTRs), a number of “endogenized” retroviral genes encode functional proteins. Previous studies have underlined the implication of these ERV-derived proteins in the development and the function of the placenta. In this review, we summarize recent findings showing that two ERV genes, termed Syncytin-1 and Syncytin-2, which encode former envelope (Env) proteins, trigger fusion events between villous cytotrophoblasts and the peripheral multinucleated syncytiotrophoblast layer. Such fusion events maintain the stability of this latter cell structure, which plays an important role in fetal development by the active secretion of various soluble factors, gas exchange and regulation of fetomaternal immunotolerance. We also highlight new studies showing that these ERV proteins, in addition to their localization at the cell surface of cytotrophoblasts, are also incorporated on the surface of various extracellular microvesicles, including exosomes. Such exosome-associated proteins could be involved in the various functions attributed to these vesicles and could provide a form of tropism. Additionally, through their immunosuppressive domains, these ERV proteins could also contribute to fetomaternal immunotolerance in a local and more distal manner. These various aspects of the implication of Syncytin-1 and -2 in placental function are also addressed in the context of the placenta-related disorder, preeclampsia.

## 1. Introduction

It is now well established that viral relics, named endogenous retroviruses, derived from ancestral infectious retroviruses, have made an important entry in vertebrates through their germ cells [1,2]. The similarities of their genomic structure (consisting of *gag*, *pro*, *pol* and *env* genes flanked by two long terminal repeats (LTRs)) with known retroviruses were thus an essential clue for their initial identification. Furthermore, upon infection and integration in germ cells, resulting proviral DNAs likely further expanded their copy number by reinfection of germ cells [1]. Through evolution, the loss of a functional Env protein by mutation (insertions, deletions, substitutions) and/or epigenetic modifications of the locus have potentially rendered these retroviral sequences unable to produce infectious virions, although retrotransposition might have led to the further increase in their copy number [1,3]. 

Although the exact nature and function of these sequences remain largely unknown, recent studies on ERVs in humans (formerly termed HERVs) have provided intriguing mechanisms of action for some of the encoding genes. ERV sequences represent up to 8% of our genome and are largely composed of solo LTRs (90%), resulting from recombination events between these flanking elements. These ERVs are classified into different families, which have been reordered in three groups based on homology [4,5,6]. Families of ERVs are normally identified with a letter, corresponding to the specific amino acid anchored to the tRNA required for the initial first strand DNA synthesis in the retrotranscription step of retroviral replication (for example, the identification of ERVW-1 is based on the tryptophan (W) amino acid attached to the tRNA, which was formally needed for retrotranscription of its genomic RNA). Interestingly, sequence analysis of several ERV sequences also revealed that typical retroviral ORFs are still present in certain loci, although they have acquired several mutations during evolution. In fact, no ERVs, including the most recently acquired ERV-K members, have been shown to be replication competent [7,8,9]. Despite the fact that ERVs have been linked to various diseases, such as multiple sclerosis, cancer and diabetes [10,11,12,13], the retention of their genes during evolution suggests that they have provided a beneficial role to human survival. In this respect, studies that have highlighted a strong implication of ERV genes in the development and the function of the placenta represent the best example of their beneficial nature. This review will focus on some of the recent findings on the association between ERVs and this important organ.

## 2. ERVs and Placenta Development

A number of studies have highlighted the implication of various ERV genes in normal placenta development. The human placenta, an indispensable organ for intrauterine fetal growth, is composed of various cell types. These cells include extravillous and villous cytotrophoblasts, the latter capable of further differentiation into an overlaying structure, known as the syncytiotrophoblast. This cell layer is a multinucleated cellular barrier, which is in direct contact with maternal blood. The syncytiotrophoblast plays a fundamental role through the optimization of the proper exchange of nutrients and hormones between the mother and the fetus and through the production of important soluble factors, such as human chorionic gonadotropin (hCG) and human placental lactogen (hPL) [14,15,16]. Additionally, the syncytiotrophoblast maintains fetomaternal tolerance by continuous interaction and monitoring of surrounding dendritic cells, macrophages, T-lymphocytes and natural killer cells. This constant monitoring allows a firm regulation of the immunosuppressive state that is absolutely required to prevent fetal rejection [17,18,19]. The placenta, and particularly, residing cytotrophoblasts, actively expresses a number of ERV envelope (Env) genes [20,21,22,23,24]. Based on these previous findings, a set of pivotal studies have demonstrated that two Env proteins, termed Syncytin-1 and Syncytin-2, were likely inducing fusion between underlying villous cytotrophoblasts and the syncytiotrophoblast layer, thereby contributing to the constant renewal and stability of this highly dynamic structure [20,22,25,26,27]. Syncytin-1 and Syncytin-2 proteins are encoded by two different ERV loci, *i.e.*, ERVW-1 and ERVFRD-1, which are located on chromosome 7 and 6, respectively. Although both proteins are expressed in the placenta, certain differences exist regarding their localization and expression patterns. In fact, expression of Syncytin-1 is mostly limited to villous and extravillous cytotrophoblasts [22,28,29,30]. A number of studies have thereby addressed the mechanism of the regulation of Syncytin-1 expression and have led to the characterization of its promoter, which is partly embedded in the 5’ LTR and is dependent on the transcription factor GCM1 along with others, such as Sp1 and GATA family members [31,32,33,34,35]. In addition, DNA methylation and histone H3K9 trimethylation at the Syncytin-1 locus have been reported to be important epigenetic modifications that silence ERVW-1 expression [36,37,38,39]. Another report has further indicated that splicing could be an alternative mechanism of the regulation of Syncytin-1 expression [40]. Similarly to other retroviral envelope proteins, Syncytin-1 interacts with receptors to mediate fusion. Indeed, conclusive reports have identified two sodium-dependent neutral amino acid transporters, namely solute carrier family 1 members 4 and 5 (SLC1A4 and SLC1A5) (otherwise known as ASCT1 and ASCT2), as its receptors [41,42,43].

However, a certain controversy has been underscored as to the role of Syncytin-1 in trophoblast fusion and its cellular distribution, which shows variation in terms of its localization in different trophoblast cell populations [44]. The discovery of Syncytin-2 has been an important finding, which has further shed light on the complexity of trophoblast fusion [20,45]. Our team has, in fact, demonstrated that Syncytin-2 played a more decisive role in cytotrophoblast fusion, when compared to Syncytin-1 [23]. Indeed, transfection experiments of siRNA in the human BeWo cell line and in primary cytotrophoblasts showed a more pronounced decrease in fusion events upon repression of Syncytin-2 expression in comparison to the conditions in which Syncytin-1 was silenced. Syncytin-2 expression is GCM-1-dependent and is specifically expressed in villous cytotrophoblasts, although we have shown an increase of its expression upon differentiation of primary cytotrophoblasts in cell culture [23,45,46]. Syncytin-2 has been associated with a single receptor, the major facilitator superfamily domain containing 2a (MFSD2a), which belongs to the carbohydrate carrier family and has recently been associated with the transport of the essential fatty acid, docosahexaenoic acid [47,48]. MFSD2a is forskolin-inducible, is expressed in the syncytiotrophoblast and has been confirmed to be implicated in fusion [48,49]. In addition to Syncytin-1 and -2, a subsequent study by Blaise *et al.* also showed that two other ERV envelope proteins, EnvV and EnvP(b), are also expressed in the placenta [21]. Interestingly, the EnvP(b) protein has retained its fusogenic potential, although being less specifically expressed in the placenta. However, using the trophoblast-like BeWo cell line model, results from our team did not support a role for this Env protein in trophoblast fusion [50]. As for EnvV, two loci have been identified, namely ERVV-1 and ERVV-2 [51], resulting from gene conversion. Recent reports suggest that one of these loci has lost its fusogenic activity in humans, but is fusogenic in placenta of Old World monkeys [52]. From these various results, it should also be underscored that in addition to the *envV* gene potentially associated with placentation in Old World monkeys, a number of *env* genes designated as Syncytin or Syncytin-like, have been strongly suggested to bear a specific role for normal placenta development in a wide variety of mammalian representatives, such as mouse, ruminants, sheep, dogs and cats [53,54,55,56,57,58,59,60,61,62,63]. This is a clear demonstration of convergent evolution and greatly contributes to our understanding of the importance of ERVs in mammalian evolution. 

**Figure 1:**
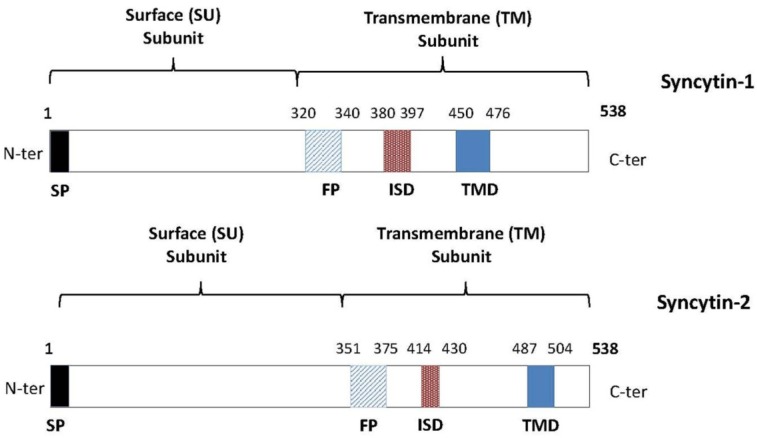
Schematic presentation of the functional domains of Syncytin-1 and -2. Similarly to other retroviral glycoproteins, Syncytin-1 and Syncytin-2 are synthesized as inactive precursors, which are then cleaved into two functional subunits: the SU and the TM subunit. SU is responsible for receptor binding and TM mediates the fusion. Both proteins are 538 amino acids long and harbor a fusion peptide (FP), a functional immunosuppressive domain (ISD) and a transmembrane domain (TMD) in their TM subunit. As a membrane protein, the polyprotein also possesses a cleaved signal peptide (SP) at its amino end.

Syncytin proteins are typical retroviral-like envelope proteins and have retained the general structure of these glycoproteins (Figure 1) [64]. Even though they originate from exogenous retroviruses having infected primate ancestors over several million years ago, these proteins have remarkably conserved their fusogenic potential. As depicted in Figure 1, both Syncytin-1 and -2 are synthesized as polyproteins, which are cleaved into surface (SU) and transmembrane (TM) subunits by the cellular furin protease [65,66].

The SU component of the envelope protein is required for receptor recognition, and the receptor-binding domain has been mapped to the NH_2_ end of Syncytin-1 [67]. The TM subunit anchors the whole envelope glycoprotein complex to the membrane through its transmembrane domain (TMD) and is directly responsible for membrane fusion between target cells and Syncytin-expressing cells upon insertion of its fusion peptide (FP) to the plasma membrane. Further studies have also revealed the functional resemblance of Syncytin-1 and -2 in comparison to common exogenous retroviruses, with a need to form trimers for proper fusion [66,68]. As an additional interesting feature of Syncytin proteins, again shared with a certain number of exogenous retroviruses, the presence of an immunosuppressive domain (ISD) has also been demonstrated in these ERV proteins [69]. 

## 3. Syncytin-1 and Syncytin-2: Potential Mediators of Immune Tolerance

Due to the presence of their ISD domain, early identification of both Syncytin-1 and-2 suggested that these proteins could be involved in the fetomaternal tolerance state prevailing during pregnancy. It is indeed known that exogenous retroviruses frequently induce severe immunosuppression in both human and animals [70]. Although the exact molecular mechanisms and interacting partners involved in the modulation of the immune response are not known, retroviral envelope proteins are likely mediators of this immune dysfunction, which depends on the presence of the immunosuppressive domain, as recently illustrated by Schlecht-Louf *et al.* with Friend murine leukemia virus (F-MLV) and a feline leukemia virus (FeLV) vaccine [71,72]. *In vitro* and *in vivo* studies have in fact shown that a synthetic retroviral 17 amino acid peptide representing the ISD is extremely immunosuppressive [69,73,74,75]. Moreover, contrary to a previous report [76], we showed that the endogenous retroviral envelope Syncytin-1 inhibits LPS/PHA-stimulated cytokine responses in human blood cells. This report suggested that Syncytin-1 is immunosuppressive and may equally be relevant to maternal immunotolerance [77]. More studies are needed to better appreciate the role of Syncytin-1 and -2 in the regulation of the immune response in the vicinity of the fetus. However, recent findings showing that these proteins are associated with various types of extracellular microvesicles are now helping to provide more adapted mechanisms of action of these proteins toward immunotolerance [77,78,79].

## 4. Exosomes and the Placenta

The placenta releases extracellular microvesicles of different types, which include exosomes and syncytiotrophoblast microparticles [80,81,82,83]. Research on exosomes, most notably in the field of placenta research, has been increasing in importance over recent years and has demonstrated that these vesicles are involved in many different normal and pathological processes. Exosome-associated proteins mediate different exosomal functions, such as intercellular communication, induced cell signaling and miRNA-dependent modulation of gene expression. Recent findings suggest that incorporated ERV Env proteins are also playing an active role [77,84].

Exosomes are part of a growing list of cellular microvesicles, including microvesicles shedding from the plasma membrane and apoptotic blebs. Originally described in rat and sheep reticulocytes [85,86,87,88], they were first functionally associated with the disposal of unnecessary proteins. It is now well established that the biological function of such microvesicles is oriented towards cell-to-cell communication. Exosomes are released by a large range of cells, including immune cells [89,90,91], neural cells [92,93], stem cells [94,95], placenta cells [77,84,96,97] and many cancer cells [98], and can be isolated from different body fluids, such as serum, urine, cerebrospinal fluid and amniotic fluid (reviewed in [99]). Several characteristics allow the distinction of exosomes from the other cellular microvesicles. Firstly, exosomes are microvesicles (40 nm–100 nm) that follow the endocytic pathway instead of directly budding from the plasma membrane. Secondly, they have an homogenous cup-shaped structure, when observed by electron microscopy, Finally, their buoyant density ranges between 1.13 g/mL and 1.19 g/mL on a sucrose gradient [100]. Exosomes are generated as intraluminal vesicles (ILVs) contained in a subtype of late endosomes called multivesicular bodies (MVBs). The general process leading to the formation of exosomes can be summarized in three major steps. Firstly, the inward budding of the membrane of late endosomes leads to the formation of ILVs within MVBs. Secondly, the newly generated MVBs can either fuse with lysosomes, thus leading to the degradation of their content, or be directed to the plasma membrane [101]. Thirdly, the MVB membrane fuses with the plasma membrane, thus allowing exosome secretion. Although the protein composition of exosomes varies according to their originating cell, proteome analyses have highlighted the presence of constitutive proteins that belong to late endosome/MVB compartments. Indeed, the tetraspanins, CD9, CD63, CD81, and the ESCRT-related proteins, Alix and TSG101, are constituents of nearly all exosomes and are markers used for the detection of exosomes [91,99,102]. The acetylcholine esterase (AChE) activity has also been associated with exosomes in several studies and is a useful tool to control for exosome isolation and purification [84,88,103,104]. 

**Figure 2:**
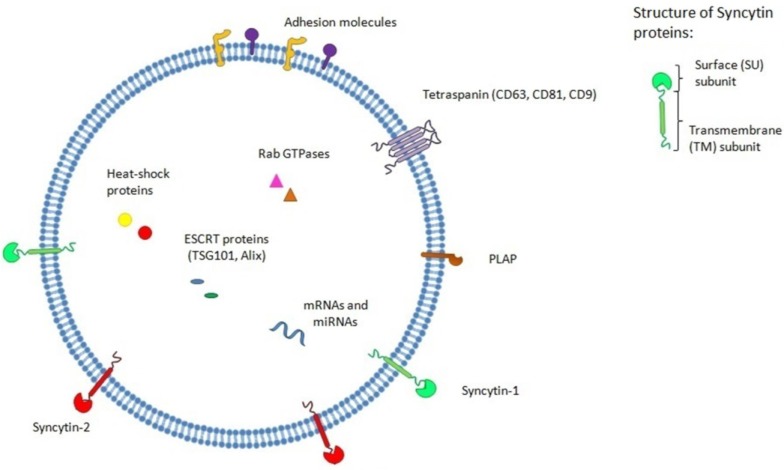
Syncytin-1 and -2 are present at the surface of placental exosomes. Schematic representation of a human placental exosome harboring Syncytin proteins on its surface. Both incorporated Syncytin-1 and -2 are composed of SU and TM subunits. The figure also depicts a certain number of proteins and RNA species most commonly associated with placental exosomes.

Exosome functions depend on their protein and RNA content and operate through direct contact with surface proteins of target cells or through modification of cell signaling or cellular gene expression upon fusion. This is particularly relevant, as exosome-associated miRNAs have the potential to alter the gene expression of targeted cells and thereby impact cell fate. In fact, many mRNA and miRNA have been isolated from different cell- or body fluid-derived exosomes, and a database is now available that combines all of the data currently available on the protein/RNA/lipid composition of exosomes (http://www.exocarta.org) [105]. More specifically, in relation to the placenta, a number of reports have focused on the miRNA and protein content of placenta-derived exosomes and have shown a very complex, yet partly placenta-selective, miRNomic profile [97]. Recent findings have further revealed that the placenta-specific miRNA cluster, C19MC, was a component of primary trophoblast-derived exosomes [106]. These latter miRNAs are likely to contribute to exosome function and, in fact, have recently been suggested to limit viral infection on recipient cells in an autophagy-dependent manner [107,108]. Exosomes originating from different placental cell types have also been reported to act on endothelial cell and smooth muscle cell migration, although the precise mechanism remains to be determined [109,110,111]. Despite these interesting findings, no clear clues as to how these exosomes are able to deliver their content to cell targets are currently available. Our team has recently suggested a mechanism of delivery, whereby ERV Env proteins incorporated at the surface of cytotrophoblast-derived exosomes bind to their specific receptors [84]. In fact, an association between viruses and exosomes has been previously described for many viral families, including ERVs [112]. In our study, we have demonstrated that exosomes isolated from the culture supernatant of primary villous cytotrophoblasts or from the serum of pregnant women harbored both Syncytin-1 and -2 (Figure 2).

**Figure 3:**
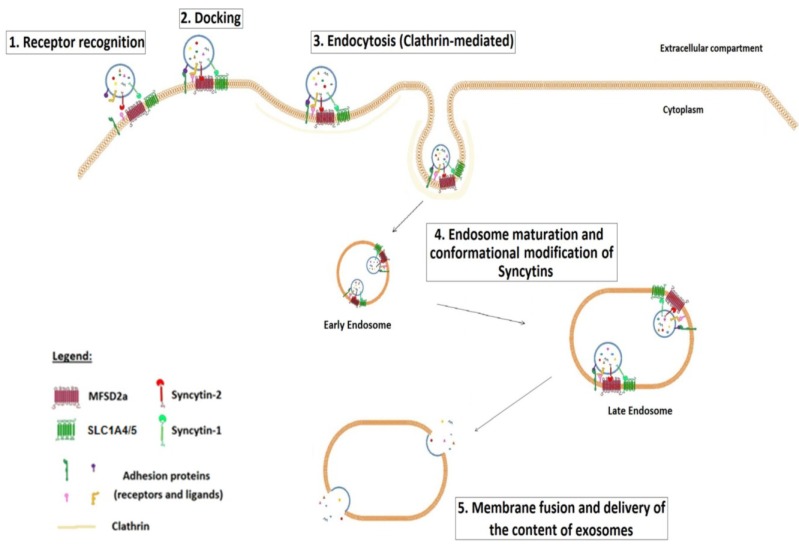
Exosome-associated Syncytin-1 and -2 allow specific entry of exosomes into target cells. Exosomes that harbor Syncytin-1 and -2 specifically target SCL1A4/5- and/or MFSD2a-expressing cells. The recognition and binding of Syncytins to their specific receptor, probably in association with other adhesion proteins (Steps 1 and 2), induce exosome endocytosis (Step 3), being potentially clathrin-mediated. Then, endosome maturation (from early to late endosomes) might bring a conformational change of Syncytins, thereby provoking fusion between exosomal and endosomal membranes (4 and 5). The exosome content is subsequently released in the cytoplasm of target cells, leading to cellular alteration.

The incorporation of Syncytin-1 and -2 at the surface of placental exosomes is also in line with our previous findings showing the important intracellular distribution of Syncytin-1 and Syncytin-2 in addition to their expected plasma membrane localization in both stimulated BeWo cells and primary cytotrophoblasts [23]. In our report on exosomes, our results further indicated that depletion of either Syncytin-1 or -2 reduced the uptake of resulting exosomes by BeWo cells. These data thus suggest that both Syncytin-1 and -2 are involved in the uptake of placental exosomes by target cells after binding to their respective receptor followed by entry through the endocytic pathway. Several adhesion proteins and ligands, such as integrins, annexins, Claudin-1 and ICAM-1, are found at the surface of exosomes and most likely contribute to the binding of exosomes to the cell surface. However, the delivery of exosome content implies the fusion of the exosome membrane with the endocytic vesicle, and in this perspective, Syncytin-1 and -2 might make a crucial contribution toward this process. Thus, we are proposing a model by which, through incorporating Syncytin proteins, placental exosomes first contact specific (SCL1A4/5 and/or MFSD2a) receptors with potential co-receptor binding mediated by other cell surface complexes (Figure 3). Exosomes are then internalized in target cells through a clathrin-coated endocytic process. The exosome-associated Syncytin-1 and -2 bound to their respective receptors could then mediate fusion with the endosomal membrane, thereby specifically delivering their content to these cells. This Env protein-dependent interaction between exosomes and target cells could thus refer to a form of exosome tropism, which is comparable to the combination of specific receptors and co-receptors that allows the infection of specific target cells by viruses. Although more experiments are needed to discern the exact mechanism underlying the functional role of these ERV Env proteins in the intracellular trafficking of internalized placental exosomes, it is clear that they potentially provide a selective interaction with target cells, which could include endothelial cells and villous/extravillous cytotrophoblasts.

## 5. Placenta Exosomes, Syncytin and Modulation of the Immune Response

The placental exosome can also interact with different immune cell populations and alter their function and activation state [82,83]. One of the early studies had demonstrated that placental exosomes modulated T-cell signaling and that the extent of repression, measured by CD3ζ- and Jak3-reduced expression, correlated with the abundance of FasL at the surface of exosomes [113,114]. Other studies have also revealed that incorporated FasL and TRAIL are activated at the surface of placental exosomes and induce apoptosis of T-cell lines and activated peripheral blood mononuclear cells (PBMC) [115]. Other examples demonstrating an effect of placental exosomes on immune response include the reduction of the cytotoxic function of NK and CD8^+^ T-cells mediated by incorporated NKG2D ligands and the impact on monocyte recruitment, macrophage differentiation and cytokine production [96,116,117,118]. As highlighted above, we have demonstrated that Syncytin-1 and -2 are incorporated on the surface of placental exosomes and could actively act in a local or distal environment on the immune response [77,84]. In fact, Holder *et al.* [78] have also showed that placental exosomes alter PBMC activation, presumably through associated Syncytin-1 [119]. Hence, in a similar manner to the exosome-cell interactions described above, placental exosomes could also interact with various immune cell types and, through Syncytin-1 and -2 ISD, be involved in the immunosuppressive state, leading to immunotolerance. It will be important to further understand how Syncytin-1 and -2 incorporated in trophoblast-derived exosomes may mediate such immunosuppression, *i.e.*, whether NK and CD8^+^ T-cells are being targeted, as suggested for the immunosuppression mediated by the Friend-MuLV envelope protein [71]. In addition, a more comprehensive mechanism of action of ISD on immune cell activation with respect to the exact nature of the cell membrane protein interacting with this domain would be valuable information.

## 6. Association between Downregulation of Syncytin-1 and -2 and Preeclampsia and Their Use as New Potential Biomarkers

Pre-eclampsia (PE) is a pregnancy disorder associated with a defect in placentation [120,121]. PE is one of the leading causes of maternal and neonatal mortality and morbidity. As appropriate management tools and preventives therapies are needed to achieve proper clinical follow-up of predisposed pregnant women, major efforts are ongoing to identify biomarkers for the early diagnosis of PE. We and others have reported that the expression of Syncytin-1 and -2 is reduced in the placental tissue of PE when compared to the tissue of normal pregnant women [65,122,123,124,125,126,127,128,129,130]. As PE has been associated with abnormal placentation (*i.e.*, through the reduced size of the syncytiotrophoblast layer) and with an exacerbated inflammatory response, reduced Syncytin-1 and -2 levels might be determinant in these PE-associated features. Furthermore, based on our recent findings that Syncytin-2 is also reduced in serum-derived exosomes from PE patients [84], we are suggesting that exosome-associated Syncytin-2 levels modify their ability to communicate with neighboring or more distant cells, such as endothelial cells, cytotrophoblasts and various immune cell population. This would, in turn, potentially impact normal placental development function and could have detrimental effects on fetomaternal immunotolerance. As a final point, reduced Syncytin-2 levels on the surface of serum-derived PE exosomes from second and third trimester samples warrants further investigation to determine if these exosomes harvested at an earlier time point (first trimester) could be an important biological material to monitor pregnant women for predisposition toward PE through associated Syncytin-2 protein levels.

## 7. Conclusions

ERV envelope proteins Syncytin-1 and -2 have been clearly shown to be implicated in normal placenta function through their fusogenic ability to drive cytotrophoblast fusion to the syncytiotrophoblast layer. Through their active immunosuppression domain, these proteins might also modulate the immune response, which otherwise would harm the fetus. Recent findings have now shed light on how these proteins could also act more distally from the placenta through their incorporation in placental exosomes. Incorporated Syncytin proteins could also affect other exosomal functions and be implicated in intercellular communication with other cell types. ERV Syncytin-1 and -2 are also importantly reduced in PE patients, and their implication at the cellular and exosomal levels could have an important consequence in relation to exosomal function related to induced immunosuppression and other placental functions. In addition, the association between Syncytin proteins and placental exosomes opens up the possible use of these proteins as markers of various obstetric disorders, such as pre-eclampsia. In conclusion, more studies are needed to mechanistically address the exact function of these intriguing proteins in placental function, and exciting developments in this area of placenta research are expected to emerge in the upcoming years.

## References

[1] Dewannieux M., Heidmann T. (2013). Endogenous retroviruses: Acquisition, amplification and taming of genome invaders. Curr. Opin. Virol..

[2] Stoye J.P. (2012). Studies of endogenous retroviruses reveal a continuing evolutionary saga. Nat. Rev. Microbiol..

[3] Magiorkinis G., Gifford R.J., Katzourakis A., de Ranter J., Belshaw R. (2012). Env-less endogenous retroviruses are genomic superspreaders. Proc. Natl. Acad. Sci. USA.

[4] Larsson E., Andersson G. (1998). Beneficial role of human endogenous retroviruses: Facts and hypotheses. Scand. J. Immunol..

[5] Blikstad V., Benachenhou F., Sperber G.O., Blomberg J. (2008). Evolution of human endogenous retroviral sequences: A conceptual account. Cell. Mol. Life Sci..

[6] Kurth R., Bannert N. (2010). Beneficial and detrimental effects of human endogenous retroviruses. Int. J. Cancer J. Int. Cancer.

[7] Blomberg J., Benachenhou F., Blikstad V., Sperber G., Mayer J. (2009). Classification and nomenclature of endogenous retroviral sequences (ervs): Problems and recommendations. Gene.

[8] Belshaw R., Dawson A.L., Woolven-Allen J., Redding J., Burt A., Tristem M. (2005). Genomewide screening reveals high levels of insertional polymorphism in the human endogenous retrovirus family herv-k(hml2): Implications for present-day activity. J. Virol..

[9] Subramanian R.P., Wildschutte J.H., Russo C., Coffin J.M. (2011). Identification, characterization, and comparative genomic distribution of the herv-k (hml-2) group of human endogenous retroviruses. Retrovirology.

[10] Antony J.M., van Marle G., Opii W., Butterfield D.A., Mallet F., Yong V.W., Wallace J.L., Deacon R.M., Warren K., Power C. (2004). Human endogenous retrovirus glycoprotein-mediated induction of redox reactants causes oligodendrocyte death and demyelination. Nat. Neurosci..

[11] Lower R., Lower J., Kurth R. (1996). The viruses in all of us: Characteristics and biological significance of human endogenous retrovirus sequences. Proc. Natl. Acad. Sci. USA.

[12] Wang-Johanning F., Frost A.R., Jian B., Azerou R., Lu D.W., Chen D.T., Johanning G.L. (2003). Detecting the expression of human endogenous retrovirus e envelope transcripts in human prostate adenocarcinoma. Cancer.

[13] Wang-Johanning F., Frost A.R., Jian B., Epp L., Lu D.W., Johanning G.L. (2003). Quantitation of herv-k env gene expression and splicing in human breast cancer. Oncogene.

[14] Muyan M., Boime I. (1997). Secretion of chorionic gonadotropin from human trophoblasts. Placenta.

[15] Handwerger S., Freemark M. (2000). The roles of placental growth hormone and placental lactogen in the regulation of human fetal growth and development. J. Pediatr. Endocrinol. Metab..

[16] Lacroix M.C., Guibourdenche J., Frendo J.L., Pidoux G., Evain-Brion D. (2002). Placental growth hormones. Endocrine.

[17] Munoz-Suano A., Hamilton A.B., Betz A.G. (2011). Gimme shelter: The immune system during pregnancy. Immunol. Rev..

[18] Nakamura O. (2009). Children's immunology, what can we learn from animal studies (1): Decidual cells induce specific immune system of feto-maternal interface. J. Toxicol. Sci..

[19] Warning J.C., McCracken S.A., Morris J.M. (2011). A balancing act: Mechanisms by which the fetus avoids rejection by the maternal immune system. Reproduction.

[20] Blaise S., de Parseval N., Benit L., Heidmann T. (2003). Genomewide screening for fusogenic human endogenous retrovirus envelopes identifies syncytin 2, a gene conserved on primate evolution. Proc. Natl. Acad. Sci. USA.

[21] Blaise S., de Parseval N., Heidmann T. (2005). Functional characterization of two newly identified human endogenous retrovirus coding envelope genes. Retrovirology.

[22] Mi S., Lee X., Li X., Veldman G.M., Finnerty H., Racie L., LaVallie E., Tang X.Y., Edouard P., Howes S. (2000). Syncytin is a captive retroviral envelope protein involved in human placental morphogenesis. Nature.

[23] Vargas A., Moreau J., Landry S., LeBellego F., Toufaily C., Rassart E., Lafond J., Barbeau B. (2009). Syncytin-2 plays an important role in the fusion of human trophoblast cells. J. Mol. Biol..

[24] Kammerer U., Germeyer A., Stengel S., Kapp M., Denner J. (2011). Human endogenous retrovirus k (herv-k) is expressed in villous and extravillous cytotrophoblast cells of the human placenta. J. Reprod. Immunol..

[25] Blond J.L., Beseme F., Duret L., Bouton O., Bedin F., Perron H., Mandrand B., Mallet F. (1999). Molecular characterization and placental expression of herv-w, a new human endogenous retrovirus family. J. Virol..

[26] Blond J.L., Lavillette D., Cheynet V., Bouton O., Oriol G., Chapel-Fernandes S., Mandrand B., Mallet F., Cosset F.L. (2000). An envelope glycoprotein of the human endogenous retrovirus herv-w is expressed in the human placenta and fuses cells expressing the type d mammalian retrovirus receptor. J. Virol..

[27] Frendo J.L., Olivier D., Cheynet V., Blond J.L., Bouton O., Vidaud M., Rabreau M., Evain-Brion D., Mallet F. (2003). Direct involvement of herv-w env glycoprotein in human trophoblast cell fusion and differentiation. Mol. Cell. Biol..

[28] Hayward M.D., Potgens A.J., Drewlo S., Kaufmann P., Rasko J.E. (2007). Distribution of human endogenous retrovirus type w receptor in normal human villous placenta. Pathology.

[29] Muir A., Lever A.M., Moffett A. (2006). Human endogenous retrovirus-w envelope (syncytin) is expressed in both villous and extravillous trophoblast populations. J. Gen. Virol..

[30] Malassine A., Handschuh K., Tsatsaris V., Gerbaud P., Cheynet V., Oriol G., Mallet F., Evain-Brion D. (2005). Expression of herv-w env glycoprotein (syncytin) in the extravillous trophoblast of first trimester human placenta. Placenta.

[31] Cheng Y.H., Handwerger S. (2005). A placenta-specific enhancer of the human syncytin gene. Biol. Reprod..

[32] Cheng Y.H., Richardson B.D., Hubert M.A., Handwerger S. (2004). Isolation and characterization of the human syncytin gene promoter. Biol. Reprod..

[33] Yu C., Shen K., Lin M., Chen P., Lin C., Chang G.D., Chen H. (2002). Gcma regulates the syncytin-mediated trophoblastic fusion. J. Biol. Chem..

[34] Chang C.W., Chang G.D., Chen H. (2011). A novel cyclic amp/epac1/camki signaling cascade promotes gcm1 desumoylation and placental cell fusion. Mol. Cell. Biol..

[35] Prudhomme S., Oriol G., Mallet F. (2004). A retroviral promoter and a cellular enhancer define a bipartite element which controls env ervwe1 placental expression. J. Virol..

[36] Zhuang X.W., Li J., Brost B.C., Xia X.Y., Chen H.B., Wang C.X., Jiang S.W. (2014). Decreased expression and altered methylation of syncytin-1 gene in human placentas associated with preeclampsia. Curr. Pharm. Des..

[37] Matouskova M., Blazkova J., Pajer P., Pavlicek A., Hejnar J. (2006). Cpg methylation suppresses transcriptional activity of human syncytin-1 in non-placental tissues. Exp. Cell Res..

[38] Li F., Nellaker C., Sabunciyan S., Yolken R.H., Jones-Brando L., Johansson A.S., Owe-Larsson B., Karlsson H. (2014). Transcriptional derepression of the ervwe1 locus following influenza a virus infection. J. Virol..

[39] Gimenez J., Montgiraud C., Oriol G., Pichon J.P., Ruel K., Tsatsaris V., Gerbaud P., Frendo J.L., Evain-Brion D., Mallet F. (2009). Comparative methylation of ervwe1/syncytin-1 and other human endogenous retrovirus ltrs in placenta tissues. DNA Res.: Int. J. Rapid Publ. Rep. Genes Genomes.

[40] Trejbalova K., Blazkova J., Matouskova M., Kucerova D., Pecnova L., Vernerova Z., Heracek J., Hirsch I., Hejnar J. (2011). Epigenetic regulation of transcription and splicing of syncytins, fusogenic glycoproteins of retroviral origin. Nucleic Acids Res..

[41] Kudo Y., Boyd C.A. (2002). Changes in expression and function of syncytin and its receptor, amino acid transport system b(0) (asct2), in human placental choriocarcinoma bewo cells during syncytialization. Placenta.

[42] Marin M., Lavillette D., Kelly S.M., Kabat D. (2003). N-linked glycosylation and sequence changes in a critical negative control region of the asct1 and asct2 neutral amino acid transporters determine their retroviral receptor functions. J. Virol..

[43] Sommerfelt M.A. (1999). Retrovirus receptors. J. Gen. Virol..

[44] Huppertz B., Bartz C., Kokozidou M. (2006). Trophoblast fusion: Fusogenic proteins, syncytins and adams, and other prerequisites for syncytial fusion. Micron.

[45] Malassine A., Blaise S., Handschuh K., Lalucque H., Dupressoir A., Evain-Brion D., Heidmann T. (2007). Expression of the fusogenic herv-frd env glycoprotein (syncytin 2) in human placenta is restricted to villous cytotrophoblastic cells. Placenta.

[46] Liang C.Y., Wang L.J., Chen C.P., Chen L.F., Chen Y.H., Chen H. (2010). Gcm1 regulation of the expression of syncytin 2 and its cognate receptor mfsd2a in human placenta. Biol. Reprod..

[47] Nguyen L.N., Ma D., Shui G., Wong P., Cazenave-Gassiot A., Zhang X., Wenk M.R., Goh E.L., Silver D.L. (2014). Mfsd2a is a transporter for the essential omega-3 fatty acid docosahexaenoic acid. Nature.

[48] Esnault C., Priet S., Ribet D., Vernochet C., Bruls T., Lavialle C., Weissenbach J., Heidmann T. (2008). A placenta-specific receptor for the fusogenic, endogenous retrovirus-derived, human syncytin-2. Proc. Natl. Acad. Sci. USA.

[49] Toufaily C., Vargas A., Lemire M., Lafond J., Rassart E., Barbeau B. (2013). Mfsd2a, the syncytin-2 receptor, is important for trophoblast fusion. Placenta.

[50] Vargas A., Thiery M., Lafond J., Barbeau B. (2012). Transcriptional and functional studies of human endogenous retrovirus envelope envp(b) and envv genes in human trophoblasts. Virology.

[51] Kjeldbjerg A.L., Villesen P., Aagaard L., Pedersen F.S. (2008). Gene conversion and purifying selection of a placenta-specific erv-v envelope gene during simian evolution. BMC Evol. Biol..

[52] Esnault C., Cornelis G., Heidmann O., Heidmann T. (2013). Differential evolutionary fate of an ancestral primate endogenous retrovirus envelope gene, the envv syncytin, captured for a function in placentation. PLoS Genet..

[53] Lavialle C., Cornelis G., Dupressoir A., Esnault C., Heidmann O., Vernochet C., Heidmann T. (2013). Paleovirology of 'syncytins', retroviral env genes exapted for a role in placentation. Philos. Trans. R. Soc. Lon. Ser. B Biol. Sci..

[54] Chuong E.B. (2013). Retroviruses facilitate the rapid evolution of the mammalian placenta. BioEssays.

[55] Dupressoir A., Marceau G., Vernochet C., Benit L., Kanellopoulos C., Sapin V., Heidmann T. (2005). Syncytin-a and syncytin-b, two fusogenic placenta-specific murine envelope genes of retroviral origin conserved in muridae. Proc. Natl. Acad. Sci. USA.

[56] Dupressoir A., Vernochet C., Bawa O., Harper F., Pierron G., Opolon P., Heidmann T. (2009). Syncytin-a knockout mice demonstrate the critical role in placentation of a fusogenic, endogenous retrovirus-derived, envelope gene. Proc. Natl. Acad. Sci. USA.

[57] Dupressoir A., Vernochet C., Harper F., Guegan J., Dessen P., Pierron G., Heidmann T. (2011). A pair of co-opted retroviral envelope syncytin genes is required for formation of the two-layered murine placental syncytiotrophoblast. Proc. Natl. Acad. Sci. USA.

[58] Heidmann O., Vernochet C., Dupressoir A., Heidmann T. (2009). Identification of an endogenous retroviral envelope gene with fusogenic activity and placenta-specific expression in the rabbit: A new "syncytin" in a third order of mammals. Retrovirology.

[59] Cornelis G., Heidmann O., Bernard-Stoecklin S., Reynaud K., Veron G., Mulot B., Dupressoir A., Heidmann T. (2012). Ancestral capture of syncytin-car1, a fusogenic endogenous retroviral envelope gene involved in placentation and conserved in carnivora. Proc. Natl. Acad. Sci. USA.

[60] Cornelis G., Heidmann O., Degrelle S.A., Vernochet C., Lavialle C., Letzelter C., Bernard-Stoecklin S., Hassanin A., Mulot B., Guillomot M. (2013). Captured retroviral envelope syncytin gene associated with the unique placental structure of higher ruminants. Proc. Natl. Acad. Sci. USA.

[61] Cornelis G., Vernochet C., Malicorne S., Souquere S., Tzika A.C., Goodman S.M., Catzeflis F., Robinson T.J., Milinkovitch M.C., Pierron G. (2014). Retroviral envelope syncytin capture in an ancestrally diverged mammalian clade for placentation in the primitive afrotherian tenrecs. Proc. Natl. Acad. Sci. USA.

[62] Redelsperger F., Cornelis G., Vernochet C., Tennant B.C., Catzeflis F., Mulot B., Heidmann O., Heidmann T., Dupressoir A. (2014). Capture of syncytin-mar1, a fusogenic endogenous retroviral envelope gene involved in placentation in the rodentia squirrel-related clade. J. Virol..

[63] Dunlap K.A., Palmarini M., Varela M., Burghardt R.C., Hayashi K., Farmer J.L., Spencer T.E. (2006). Endogenous retroviruses regulate periimplantation placental growth and differentiation. Proc. Natl. Acad. Sci. USA.

[64] Benit L., Dessen P., Heidmann T. (2001). Identification, phylogeny, and evolution of retroviral elements based on their envelope genes. J. Virol.

[65] Chen C.P., Chen L.F., Yang S.R., Chen C.Y., Ko C.C., Chang G.D., Chen H. (2008). Functional characterization of the human placental fusogenic membrane protein syncytin 2. Biol. Reprod..

[66] Cheynet V., Ruggieri A., Oriol G., Blond J.L., Boson B., Vachot L., Verrier B., Cosset F.L., Mallet F. (2005). Synthesis, assembly, and processing of the env ervwe1/syncytin human endogenous retroviral envelope. J. Virol..

[67] Cheynet V., Oriol G., Mallet F. (2006). Identification of the hasct2-binding domain of the env ervwe1/syncytin-1 fusogenic glycoprotein. Retrovirology.

[68] Gong R., Peng X., Kang S., Feng H., Huang J., Zhang W., Lin D., Tien P., Xiao G. (2005). Structural characterization of the fusion core in syncytin, envelope protein of human endogenous retrovirus family w. Biochem. Biophys. Res. Commun..

[69] Haraguchi S., Good R.A., Day-Good N.K. (2008). A potent immunosuppressive retroviral peptide: Cytokine patterns and signaling pathways. Immunol. Res..

[70] Denner J. (2014). The transmembrane proteins contribute to immunodeficiencies induced by hiv-1 and other retroviruses. AIDS.

[71] Schlecht-Louf G., Renard M., Mangeney M., Letzelter C., Richaud A., Ducos B., Bouallaga I., Heidmann T. (2010). Retroviral infection *in vivo* requires an immune escape virulence factor encrypted in the envelope protein of oncoretroviruses. Proc. Natl. Acad. Sci. USA.

[72] Schlecht-Louf G., Mangeney M., El-Garch H., Lacombe V., Poulet H., Heidmann T. (2014). A targeted mutation within the feline leukemia virus (felv) envelope protein immunosuppressive domain to improve a canarypox virus-vectored felv vaccine. J. Virol..

[73] Cianciolo G.J., Bogerd H.P., Kipnis R.J., Copeland T.D., Oroszlan S., Snyderman R. (1985). Inhibition of lymphocyte proliferation by a synthetic peptide homologous to envelope proteins of human and animal retroviruses. Trans. Assoc. Am. Physicians.

[74] Haraguchi S., Good R.A., Cianciolo G.J., Engelman R.W., Day N.K. (1997). Immunosuppressive retroviral peptides: Immunopathological implications for immunosuppressive influences of retroviral infections. J. Leukoc. Biol..

[75] Haraguchi S., Good R.A., Day N.K. (1995). Immunosuppressive retroviral peptides: Camp and cytokine patterns. Immunol. Today.

[76] Mangeney M., Renard M., Schlecht-Louf G., Bouallaga I., Heidmann O., Letzelter C., Richaud A., Ducos B., Heidmann T. (2007). Placental syncytins: Genetic disjunction between the fusogenic and immunosuppressive activity of retroviral envelope proteins. Proc. Natl. Acad. Sci. USA.

[77] Tolosa J.M., Schjenken J.E., Clifton V.L., Vargas A., Barbeau B., Lowry P., Maiti K., Smith R. (2012). The endogenous retroviral envelope protein syncytin-1 inhibits lps/pha-stimulated cytokine responses in human blood and is sorted into placental exosomes. Placenta.

[78] Holder B.S., Tower C.L., Forbes K., Mulla M.J., Aplin J.D., Abrahams V.M. (2012). Immune cell activation by trophoblast-derived microvesicles is mediated by syncytin 1. Immunology.

[79] Record M. (2014). Intercellular communication by exosomes in placenta: A possible role in cell fusion?. Placenta.

[80] Redman C.W., Sargent I.L. (2007). Microparticles and immunomodulation in pregnancy and pre-eclampsia. J. Reprod. Immunol..

[81] Redman C.W., Tannetta D.S., Dragovic R.A., Gardiner C., Southcombe J.H., Collett G.P., Sargent I.L. (2012). Review: Does size matter? Placental debris and the pathophysiology of pre-eclampsia. Placenta.

[82] Mincheva-Nilsson L., Baranov V. (2010). The role of placental exosomes in reproduction. Am. J. Reprod. Immunol..

[83] Mincheva-Nilsson L., Baranov V. (2014). Placenta-derived exosomes and syncytiotrophoblast microparticles and their role in human reproduction: Immune modulation for pregnancy success. Am. J. Reprod. Immunol..

[84] Vargas A., Zhou S., Ethier-Chiasson M., Flipo D., Lafond J., Gilbert C., Barbeau B. (2014). Syncytin proteins incorporated in placenta exosomes are important for cell uptake and show variation in abundance in serum exosomes from patients with preeclampsia. FASEB J..

[85] Harding C., Heuser J., Stahl P. (1983). Receptor-mediated endocytosis of transferrin and recycling of the transferrin receptor in rat reticulocytes. J. Cell. Biol.

[86] Harding C., Heuser J., Stahl P. (1984). Endocytosis and intracellular processing of transferrin and colloidal gold-transferrin in rat reticulocytes: Demonstration of a pathway for receptor shedding. Eur. J. Cell Biol..

[87] Pan B.T., Johnstone R.M. (1983). Fate of the transferrin receptor during maturation of sheep reticulocytes *in vitro*: Selective externalization of the receptor. Cell.

[88] Johnstone R.M., Adam M., Hammond J.R., Orr L., Turbide C. (1987). Vesicle formation during reticulocyte maturation. Association of plasma membrane activities with released vesicles (exosomes). J. Biol. Chem..

[89] Denzer K., van Eijk M., Kleijmeer M.J., Jakobson E., de Groot C., Geuze H.J. (2000). Follicular dendritic cells carry mhc class ii-expressing microvesicles at their surface. J. Immunol..

[90] Raposo G., Nijman H.W., Stoorvogel W., Liejendekker R., Harding C.V., Melief C.J., Geuze H.J. (1996). B lymphocytes secrete antigen-presenting vesicles. J. Exp. Med..

[91] Thery C., Boussac M., Veron P., Ricciardi-Castagnoli P., Raposo G., Garin J., Amigorena S. (2001). Proteomic analysis of dendritic cell-derived exosomes: A secreted subcellular compartment distinct from apoptotic vesicles. J. Immunol..

[92] Faure J., Lachenal G., Court M., Hirrlinger J., Chatellard-Causse C., Blot B., Grange J., Schoehn G., Goldberg Y., Boyer V. (2006). Exosomes are released by cultured cortical neurones. Mol. Cell. Neurosci..

[93] Guescini M., Genedani S., Stocchi V., Agnati L.F. (2010). Astrocytes and glioblastoma cells release exosomes carrying mtdna. J. Neural Transm..

[94] Kang D., Oh S., Ahn S.M., Lee B.H., Moon M.H. (2008). Proteomic analysis of exosomes from human neural stem cells by flow field-flow fractionation and nanoflow liquid chromatography-tandem mass spectrometry. J. Proteome Res..

[95] Lai R.C., Arslan F., Lee M.M., Sze N.S., Choo A., Chen T.S., Salto-Tellez M., Timmers L., Lee C.N., El Oakley R.M. (2010). Exosome secreted by msc reduces myocardial ischemia/reperfusion injury. Stem Cell. Res..

[96] Atay S., Gercel-Taylor C., Suttles J., Mor G., Taylor D.D. (2011). Trophoblast-derived exosomes mediate monocyte recruitment and differentiation. Am. J. Reprod. Immunol..

[97] Luo S.S., Ishibashi O., Ishikawa G., Ishikawa T., Katayama A., Mishima T., Takizawa T., Shigihara T., Goto T., Izumi A. (2009). Human villous trophoblasts express and secrete placenta-specific micrornas into maternal circulation via exosomes. Biol. Reprod..

[98] Sun Y., Liu J. (2014). Potential of cancer cell-derived exosomes in clinical application: A review of recent research advances. Clin. Ther..

[99] Simpson R.J., Jensen S.S., Lim J.W. (2008). Proteomic profiling of exosomes: Current perspectives. Proteomics.

[100] Thery C., Amigorena S., Raposo G., Clayton A. (2006). Isolation and characterization of exosomes from cell culture supernatants and biological fluids. Curr. Protoc. Cell. Biol..

[101] Hanson P.I., Cashikar A. (2012). Multivesicular body morphogenesis. Annu. Rev. Cell. Dev. Biol..

[102] Atay S., Gercel-Taylor C., Kesimer M., Taylor D.D. (2011). Morphologic and proteomic characterization of exosomes released by cultured extravillous trophoblast cells. Exp. Cell Res..

[103] Cantin R., Diou J., Belanger D., Tremblay A.M., Gilbert C. (2008). Discrimination between exosomes and hiv-1: Purification of both vesicles from cell-free supernatants. J. Immunol. Methods.

[104] Savina A., Vidal M., Colombo M.I. (2002). The exosome pathway in k562 cells is regulated by rab11. J. Cell. Sci..

[105] Simpson R.J., Kalra H., Mathivanan S. (2012). Exocarta as a resource for exosomal research. J. Extracell Vesicles.

[106] Donker R.B., Mouillet J.F., Chu T., Hubel C.A., Stolz D.B., Morelli A.E., Sadovsky Y. (2012). The expression profile of c19mc micrornas in primary human trophoblast cells and exosomes. Mol. Hum. Reprod..

[107] Ouyang Y., Mouillet J.F., Coyne C.B., Sadovsky Y. (2014). Review: Placenta-specific micrornas in exosomes - good things come in nano-packages. Placenta.

[108] Delorme-Axford E., Donker R.B., Mouillet J.F., Chu T., Bayer A., Ouyang Y., Wang T., Stolz D.B., Sarkar S.N., Morelli A.E. (2013). Human placental trophoblasts confer viral resistance to recipient cells. Proc. Natl. Acad. Sci. USA.

[109] Salomon C., Ryan J., Sobrevia L., Kobayashi M., Ashman K., Mitchell M., Rice G.E. (2013). Exosomal signaling during hypoxia mediates microvascular endothelial cell migration and vasculogenesis. PLoS One.

[110] Salomon C., Torres M.J., Kobayashi M., Scholz-Romero K., Sobrevia L., Dobierzewska A., Illanes S.E., Mitchell M.D., Rice G.E. (2014). A gestational profile of placental exosomes in maternal plasma and their effects on endothelial cell migration. PLoS One.

[111] Salomon C., Yee S., Scholz-Romero K., Kobayashi M., Vaswani K., Kvaskoff D., Illanes S.E., Mitchell M.D., Rice G.E. (2014). Extravillous trophoblast cells-derived exosomes promote vascular smooth muscle cell migration. Front. Pharmacol..

[112] Wurdinger T., Gatson N.N., Balaj L., Kaur B., Breakefield X.O., Pegtel D.M. (2012). Extracellular vesicles and their convergence with viral pathways. Adv. Virol..

[113] Taylor D.D., Akyol S., Gercel-Taylor C. (2006). Pregnancy-associated exosomes and their modulation of t cell signaling. J. Immunol..

[114] Sabapatha A., Gercel-Taylor C., Taylor D.D. (2006). Specific isolation of placenta-derived exosomes from the circulation of pregnant women and their immunoregulatory consequences. Am. J. Reprod. Immunol..

[115] Stenqvist A.C., Nagaeva O., Baranov V., Mincheva-Nilsson L. (2013). Exosomes secreted by human placenta carry functional fas ligand and trail molecules and convey apoptosis in activated immune cells, suggesting exosome-mediated immune privilege of the fetus. J. Immunol..

[116] Hedlund M., Stenqvist A.C., Nagaeva O., Kjellberg L., Wulff M., Baranov V., Mincheva-Nilsson L. (2009). Human placenta expresses and secretes nkg2d ligands via exosomes that down-modulate the cognate receptor expression: Evidence for immunosuppressive function. J. Immunol..

[117] Mincheva-Nilsson L., Nagaeva O., Chen T., Stendahl U., Antsiferova J., Mogren I., Hernestal J., Baranov V. (2006). Placenta-derived soluble mhc class i chain-related molecules down-regulate nkg2d receptor on peripheral blood mononuclear cells during human pregnancy: A possible novel immune escape mechanism for fetal survival. J. Immunol..

[118] Atay S., Gercel-Taylor C., Taylor D.D. (2011). Human trophoblast-derived exosomal fibronectin induces pro-inflammatory il-1beta production by macrophages. Am. J. Reprod. Immunol..

[119] Holder B.S., Tower C.L., Abrahams V.M., Aplin J.D. (2012). Syncytin 1 in the human placenta. Placenta.

[120] Genbacev O., Joslin R., Damsky C.H., Polliotti B.M., Fisher S.J. (1996). Hypoxia alters early gestation human cytotrophoblast differentiation/invasion *in vitro* and models the placental defects that occur in preeclampsia. J. Clin. Invest..

[121] Redline R.W., Patterson P. (1995). Pre-eclampsia is associated with an excess of proliferative immature intermediate trophoblast. Hum. Pathol..

[122] Vargas A., Toufaily C., LeBellego F., Rassart E., Lafond J., Barbeau B. (2011). Reduced expression of both syncytin 1 and syncytin 2 correlates with severity of preeclampsia. Reprod. Sci..

[123] Knerr I., Huppertz B., Weigel C., Dotsch J., Wich C., Schild R.L., Beckmann M.W., Rascher W. (2004). Endogenous retroviral syncytin: Compilation of experimental research on syncytin and its possible role in normal and disturbed human placentogenesis. Mol. Hum. Reprod..

[124] Chen C.P., Wang K.G., Chen C.Y., Yu C., Chuang H.C., Chen H. (2006). Altered placental syncytin and its receptor asct2 expression in placental development and pre-eclampsia. Int. J. Obstet. Gynaecol..

[125] Keith J.C., Pijnenborg R., van Assche F.A. (2002). Placental syncytin expression in normal and preeclamptic pregnancies. Am. J. Obstet. Gynecol..

[126] Knerr I., Beinder E., Rascher W. (2002). Syncytin, a novel human endogenous retroviral gene in human placenta: Evidence for its dysregulation in preeclampsia and hellp syndrome. Am. J. Obstet. Gynecol..

[127] Kudaka W., Oda T., Jinno Y., Yoshimi N., Aoki Y. (2008). Cellular localization of placenta-specific human endogenous retrovirus (herv) transcripts and their possible implication in pregnancy-induced hypertension. Placenta.

[128] Langbein M., Strick R., Strissel P.L., Vogt N., Parsch H., Beckmann M.W., Schild R.L. (2008). Impaired cytotrophoblast cell-cell fusion is associated with reduced syncytin and increased apoptosis in patients with placental dysfunction. Mol. Reprod. Dev..

[129] Lee X., Keith J.C., Stumm N., Moutsatsos I., McCoy J.M., Crum C.P., Genest D., Chin D., Ehrenfels C., Pijnenborg R. (2001). Downregulation of placental syncytin expression and abnormal protein localization in pre-eclampsia. Placenta.

[130] Ruebner M., Strissel P.L., Ekici A.B., Stiegler E., Dammer U., Goecke T.W., Faschingbauer F., Fahlbusch F.B., Beckmann M.W., Strick R. (2013). Reduced syncytin-1 expression levels in placental syndromes correlates with epigenetic hypermethylation of the ervw-1 promoter region. PLoS One.

